# We Must Take Advantage of This Pandemic to Make a Radical Social Change: The Coronavirus as a Global Health, Inequality, and Eco-Social Problem

**DOI:** 10.1177/0020731420946594

**Published:** 2020-08-03

**Authors:** Joan Benach

**Affiliations:** 1 Health Inequalities Research Group (GREDS-EMCONET), Department of Political and Social Sciences, 16770 Universitat Pompeu Fabra, Barcelona, Spain; 2 Johns-Hopkins-UPF Public Policy Center, Barcelona, Spain; 3 GinTrans2, Madrid Autonomous University (UAM), Madrid, Spain

**Keywords:** capitalism, COVID-19, eco-social crisis, health inequalities, policy change, public health

## Abstract

COVID-19 not only constitutes a serious public health problem and a global major threat to the poorest and most vulnerable social groups and neighborhoods of the world, creating a potential pandemic of inequality, but also poses an enormous challenge from the perspective of public health, ethics, economy, environment, and politics. However, many of the deep and complex systemic interrelationships created and developed by this pandemic are largely hidden, unknown, or neglected, both by the hegemonic media and by a highly specialized and fragmented academic world. However, when all the available knowledge is critically integrated, the origins and effects underlying this pandemic are likely to be found in the development of neoliberal capitalism and its inherent logic of ceaseless accumulation, economic growth, large inequalities, and ecological devastation. This commentary reflects on these issues, drawing out some of the most important lessons to be learned and challenges to be faced in the COVID-19 pandemic and its aftermath, advocating for a radical social change to deal with these challenges.

So nature does indeed need protection from man; but man, too, needs protection from his own acts, for he is part of the living world. His war against nature is inevitably a war against himself.

– Rachel Carson^1^

Is capitalism a disease?

– Richard Levins^2^

## The Main and Immediate Public Health Concerns Associated With the Pandemic in Wealthy Countries

In high-income countries, we are in a temporary situation of “low-income-worldization” of public health care. It is shameful and scandalous to hear many reactionary and neoliberal politicians preach calm and confidence in the face of the crisis, calling health care workers “heroes” when just previously they had disparaged those very same workers by deepening the lack of resources and increasing precariousness in those jobs. From a public health point of view, in addition to its direct impact on mortality (especially severe in nursing homes) and morbidity, the pandemic is causing delays in diagnostic tests and surgical operations for hundreds of thousands of chronically ill people and in interventions for people with mental ill health, while also delaying or complicating treatment for serious or very serious health processes. From the ethical point of view, there are dilemmas about how, when, and on whom to act: Professionals must decide who can live or die, as happens with great frequency in low-income countries. From an emotional and psychological point of view, the impacts of the pandemic – including heightened stress levels and emotional trauma – on professionals, patients, and families is undoubtedly enormous. Family members cannot say goodbye to or mourn their deceased loved ones who, in many cases, cannot even be buried or cremated. The most positive aspect of this experience is the commendable commitment, bravery, and immense effort of health and social professionals and many other workers of all kinds, who were invisible before, and the huge outpouring of solidarity, community work, pride, and hope that has been triggered in many people.

## The Main Problems to Consider in the Medium and Long Term

First, long-term confinement will have a negative impact on the mental and emotional health of the population, with the highly likely emergence of outbreaks of violence related to insecurity and social changes. One example is the case of women who must confine themselves together with their abusers. Another issue is that the virus is likely to remain with us, mutate, recur, or even become more virulent, and furthermore, more severe pandemics may appear. Third, many countries do not have appropriate public health and health care systems for dealing with this coronavirus or, of course, many day-to-day illnesses, nor is there an adequate global public health system that can deal with global systemic threats similar to that of the coronavirus. Finally, we do not know when we will have an effective vaccine without side effects, but so far we do know that Big Pharma is focused on the great benefits they can reap from profitable diseases and not on tropical infections or the flu. Because there could be a fight over the vaccine monopoly, the problem then would be whether the poorer nations could have access to it.

## The Coronavirus Pandemic as a Problem of Inequality

The pandemic constitutes an enormous threat to the poorest and most vulnerable social groups and neighborhoods in many countries, living with fragile and even dire social determinants of health: poor housing; poverty; precariousness; lack of basic services, water, and food; environmental pollutants, etc. These conditions are prevalent in sub-Saharan Africa, Iran, and India, which, although relatively protected by having young populations, face a public health disaster; in the West Bank and Gaza (Occupied Palestinian Territories), which have very serious problems in terms of having to face homicidal apartheid; and for the population and poor neighborhoods of a country like the United States, where President Donald Trump’s criminal policies could generate a social catastrophe with fatal consequences for an enormous number of impoverished communities. In Western European societies, the pandemic will impact existing inequalities in a variety of ways, although we will have to wait until we have adequate studies to understand this impact in detail.

In relation to the health sector, the disproportionate risk faced by health and social services professionals who often work with scarce or inadequate resources, and by those who care for elderly women and men in nursing homes, stands out. Regarding the work environment, the most vulnerable are those who are fired from their jobs and the laborers and precarious workers whose work cannot be done from the safety of their homes, forcing them to face the dilemma of losing their jobs or getting sick. Telecommuting is a luxury not afforded to cleaners, housekeepers, care workers, cashiers, and employees in many other largely precarious and feminized occupations. Moreover, these occupations have worse social, environmental, and occupational determinants of health, all of which will further worsen individuals’ confinement conditions and, more than likely, mental health. At home, the crisis is manifested most profoundly in the women who carry the greatest burden: taking care of the elderly, sick, or disabled; children who cannot go to school; other dependent family members, etc.

Thus, COVID-19 has all of the characteristics for us to consider it not only a viral pandemic, but a “systemic pandemic of inequality” in health according to class, gender, age, ethnicity, migration status, and place of residence.

## How the Hegemonic Media Hide the Root Causes of the Pandemic

One of the great omissions of the mainstream media regards the causes of the pandemic: The common narrative of the coronavirus is as though it were a “curse” coming from abroad, which has turned into a “war” that must be seen out, and once that has happened, sooner or later, we will return to the “normality” of everyday life. Many of the deep and complex systemic interrelationships of the pandemic (ecological destruction, the economic crisis, the psychology and culture of fear, unemployment, precariousness, etc.) are largely hidden in the official account of the hegemonic media outlets, which are dominated by immediate, minute-by-minute reporting. It is logical that the media talk about the data, who is affected, and what needs to be done to get out of this crisis as soon as possible. But beyond knowing what’s going on, it is crucial to understand why things happen and try to change them.

## The Origin of the Pandemic

The underlying reason for the pandemic must be found in capitalism. The most relevant factors have to do with the global alteration of ecosystems, which is also associated with the eco-social and climatic crisis that we are experiencing. Deforestation in Southeast Asia and massive changes in land use; habitat fragmentation and excessive urbanization; the growth of a massive agro-industry; the transmission of diseases between many species in close contact with each other, which are then passed to humans as in this zoonotic virus, the SARS-CoV-2 coronavirus; the traditional diet that includes wild animals in countries such as China; the destruction of biodiversity; the massive growth of tourism and air travel; and the weakness and commodification of public health systems and social services are without doubt the most important. If we integrate and interpret all of this, we see that what lies behind is neoliberal capitalism and its inherent logic of accumulation, economic growth, profit, and inequality, which crashes against planetary biophysical limits in a “full” world.

## What This Pandemic Teaches Us in Relation to the Capitalist System in Which We Live

The first aspect to highlight concerns assessing what to do in emergencies. In the short term of an emergency situation, it is evident that all resources and efforts must be directed toward the reduction of mortality and suffering. Above all, the objectives should be saving lives, protecting families and workers, and safeguarding companies in an economy that is practically at a standstill. Unlike private services that are based on short-term profit and therefore can only organize limited emergency plans, public services can prepare and organize social emergency plans in “normal” times, making societies resilient to events such as fires, earthquakes, or pandemics.

A second lesson is to carefully assess the severity of the pandemic. There is no doubt that we are facing a very serious pandemic. However, if the current pandemic had a level of lethality equivalent to the poorly named “Spanish flu” of 1918, worldwide perhaps between 200 and 400 million would die. This pandemic also reflects our ignorance and capacity for amnesia: In 2009, the H1N1 influenza pandemic (H1N1pdm09 virus) killed between 150,000 and 575,000 people worldwide.^3^ The difference is that now, the pandemic is affecting us, the inhabitants of rich countries, more directly, and that has paralyzed the global economy. Furthermore, public health problems are abundant in the world – for example, the 140,000 deaths a year from measles, a preventable disease with a cheap and highly effective vaccine,^4^ or the more than 1.6 million annual deaths from diarrhea.^5^ The fact is that the greatest pandemic is social inequality, which creates health inequalities. Paradoxically, despite the negative effects of the coronavirus on health and the economy, the economic slowdown has certain beneficial effects for the climate and ecological crisis and other health-related phenomena. This apparent contradiction is resolved by understanding the perverse logic of an economy based in endless growth that, as it grows, destroys the material bases that sustain life, and as it decreases, improves biogeophysical indicators and various indicators of collective health.

A third lesson to be drawn is the need for a different kind of knowledge that allows us to comprehend complex historical processes. Today we have an abundance of genomic and virological knowledge, diagnostic tests, vaccine creation, epidemiology, ecology, and extensive psychological, sociological, and political knowledge, but unfortunately little integrated systemic, historical, and political knowledge. At the same time, today’s civilization has created an enormous variety of destructive processes that must be understood and modified. We live in a “full world” (i.e., one in ecological overshooting in which we are consuming 1.7 planets a year),^6^ with a huge population – almost 8,000 million human beings – living a dynamically complex existence with constant global movement. During the 14th-century plague, the pandemic moved from China to England in perhaps a decade. Now, the “tourism pandemic” entails large numbers of people moving from one side of the planet to the other in a matter of a few hours. The generation of new global epidemics must be prevented by understanding the conditions that facilitate the transmission of new viruses that may endanger all of humanity. This means avoiding the ecological destruction and social inequality that we are rapidly producing under capitalism.

The fourth lesson is that of humility. How can it be that a tiny infectious agent can generate such a global disaster, with a social and economic crisis of this magnitude? Already in the 19th century, Friedrich Engels said: “Let us not, however, flatter ourselves overmuch on account of our human conquest over nature. For each such conquest takes its revenge on us … Thus at every step we are reminded that we by no means rule over nature like a conqueror over a foreign people.”^7^ We are not invulnerable, but human beings, fragile, intra-dependent, interdependent, and eco-dependent. We are intrinsically dependent on our own psychobiology, physiological needs, and cultural environment; we are dependent on others from before birth until the moment of death; and we are dependent on nature, of which we are part and without which we cannot survive.

One last lesson to consider is of a practical and political nature. We have to change, and we have to change radically. We live in a context of fossil capitalism, with exponential growth in production and consumption, based on spending huge and cheap amounts of fossil fuels and finite natural materials that are running out. This means that we are facing not only the climatic emergency but also a major ecological crisis, which can only be understood by also understanding the energy crisis. Paraphrasing Naomi Klein when talking about the climate crisis,^8^ we can say that this pandemic changes everything, and that it is essential that we take advantage of it to create radical social change. This includes changing our individual and daily lives to move toward a more humane and truly sustainable world, by creating a homeostatic economy that uses much less primary energy and adapts its eco-social metabolism to the biophysical limits of the Earth.

## The Great Medium- and Long-Term Challenges Posed by the Coronavirus Pandemic

The economics and cultural logic of capitalism must be dismantled as soon as possible. Perhaps we will be able to cope – better or worse – with this pandemic, or with the overall conditions of the coming social and economic crisis. Perhaps we can improve equity levels, the environment, housing, or the social factors that create inequality and insecurity. Doing all this is very important, needless to say, but if we continue with the current model of exploitative capitalism, we will continue to further promote the great ecological, nuclear, biological, and political threats that will end civilization (and perhaps, in the worst case, the human species). That means there are many challenges that we must reflect on and work on. One of them is the value of the commons. This pandemic places us in a “war economy” where the value of the public and the commons, of the community, are a hope for change. Another key challenge is to generate a culture of hope, joy, and life. We must develop – from all possible angles – an understanding of how to live well: how we can live better with much less and be more together, with more emotional empathy, solidarity, and fraternity. To promote this is to promote an anti-capitalist, ecofeminist, and anti-colonial culture. Another essential challenge has to do with how to understand and transform the global and the local simultaneously. Social and critical movements generally deal with concrete issues at the local level. This is fundamental, but also insufficient in today’s world. We need to implement systemic policies, which requires analysis, a program, organization, and management. A final challenge is to understand the enemy and the adversary; to be strategic; to understand that we are in a process of brutal class struggle.

In the long term, I only see 2 main pathways: either a transformation toward the common good, or a road to barbarism and extermination. Currently, we have only had a small taste of the reactions that are likely to come from the establishment to preserve their status quo – for example, that of Dominic Cummings, senior strategist of Boris Johnson’s government in the United Kingdom, when, semi-concealed by a technical formula, he justified the U.K. policy of protecting business at the cost of many people dying.^9^ In the United States, we have witnessed Donald Trump’s statements in which he expressed his intention to buy the rights to a potential vaccine for the exclusive use of Americans.^10^ In Brazil, Jair Bolsonaro has said that COVID-19 is a “little cold,” that children and young people are not in danger, that schools should not close, and that the economy should continue as is.^11^

After the initial shock of the crisis will come the post-pandemic economic shock, and the political decisions to be made will be the “social laboratory” in which the future of humanity will be at stake. It will be a time of growing fear and insecurity, a perfect breeding ground for demagogues and neofascists. In his important book *Auschwitz, Does the 21st Century Begin? Hitler as a Precursor*,^12^ the German writer Carl Amery wrote that the struggle for scarce resources on a finite earth was the key theme of this century, warning of a world in which a superior neofascist group would want to impose their way of life on an inferior group that would be enslaved. Therefore, one possible path is the authoritarian path of total population control: a militarized society that would be characterized by social control, repression, and racism. An authoritarian techno-digital society that would bring us closer to the surveillance and control implemented in China and other Asian countries. The other general direction that we must imagine, promote, and fight for is a much more democratic society that takes care of all forms of life. COVID-19 teaches us the importance of public health and equity, and how fundamental it is to create an economy that is organized around human well-being, care of all life, and ecological stability, rather than the incessant accumulation of capital. The most powerful groups and elites of the world will not relinquish their power or their privileges; they have never done so nor will they do so. It is truly moving to see the many cooperative and social initiatives being formed today. But when the acute pandemic passes, it will be essential to have built an organized and conscious civil society to deal with the ubiquitous, systemic crisis that will follow: the “chronic pandemic.” The collective struggle must be unitary, persistent, intelligent, and extremely determined.
